# Mutation or loss of Wilms' tumor gene 1 (WT1) are not major reasons for immune escape in patients with AML receiving WT1 peptide vaccination

**DOI:** 10.1186/1479-5876-8-5

**Published:** 2010-01-21

**Authors:** Antonia Busse, Anne Letsch, Carmen Scheibenbogen, Anika Nonnenmacher, Sebastian Ochsenreither, Eckhard Thiel, Ulrich Keilholz

**Affiliations:** 1 Charité - CBF, Department of Medicine III, Berlin, Germany; 2 Institute of Medical Immunology, Charité - CCM, Berlin, Germany

## Abstract

**Background:**

Efficacy of cancer vaccines may be limited due to immune escape mechanisms like loss or mutation of target antigens. Here, we analyzed 10 HLA-A2 positive patients with acute myeloid leukemia (AML) for loss or mutations of the WT1 epitope or epitope flanking sequences that may abolish proper T cell recognition or epitope presentation.

**Methods:**

All patients had been enrolled in a WT1 peptide phase II vaccination trial (NCT00153582) and ultimately progressed despite induction of a WT1 specific T cell response. Blood and bone marrow samples prior to vaccination and during progression were analyzed for mRNA expression level of WT1. Base exchanges within the epitope sequence or flanking regions (10 amino acids N- and C-terminal of the epitope) were assessed with melting point analysis and sequencing. HLA class I expression and WT1 protein expression was analyzed by flow cytometry.

**Results:**

Only in one patient, downregulation of WT1 mRNA by 1 log and loss of WT1 detection on protein level at time of disease progression was observed. No mutation leading to a base exchange within the epitope sequence or epitope flanking sequences could be detected in any patient. Further, no loss of HLA class I expression on leukemic blasts was observed.

**Conclusion:**

Defects in antigen presentation caused by loss or mutation of WT1 or downregulation of HLA molecules are not the major basis for escape from the immune response induced by WT1 peptide vaccination.

## Background

Over-expression of Wilms' tumor gene 1 (WT1) is present in a variety of malignant tumors, including acute leukemias [1-3] and a variety of solid neoplasms [4]. The WT1 protein is a transcription factor critically involved in tumor cell proliferation, making it a suitable target for therapeutic strategies including vaccine approaches [5]. Clinical vaccination trials with WT1 peptides and protein in AML/MDS have been recently initiated leading to the induction of epitope specific cytotoxic T cells and unprecedented clinical efficacy [6-8]. However, even in case of induction of a robust T cell response cancer vaccines in general have only limited efficacy. Several immune escape mechanisms have been identified [9-11]. Important escape mechanisms on tumor cell site are loss or downregulation of tumor associated antigens (TAA) and mutation of TAA [12,13]. A mutation within the sequence of an epitope may abolish proper HLA class I binding, T cell recognition or proteasomal processing. Another less recognised mechanism interfering with antigen presentation may be a mutation of the flanking sequence of an epitope that may prohibit or decrease processing of the epitope by the proteasome or extraproteasomal proteases [14,15]. In addition, antigen presentation can be distorted by mechanisms such as decrease in HLA class I expression [16-18] or alterations in the antigen processing pathway [19-21].

Here we address loss or mutation of WT1 as a potential immune evasion mechanism in patients from a clinical phase II trial of WT1 peptide vaccination in acute myeloid leukaemia (AML).

## Methods

### Patients

Patients were treated within a phase II vaccination trial (NCT00153582) [8] and received sequential vaccinations with the HLA-A2-restricted WT1 126-134 peptide + KLH and GM-CSF as adjuvants. Detailed patient characteristics are previously published in Keilholz et al 2009: patient no 1; no 4; no 5; no 8; no 9; no 11; no 12; no 13; no 15; one patient is not published yet) [8]. All patients gave written informed consent to participate in the study according to the Declaration of Helsinki. The study was approved by the local ethics board.

### Blood and bone marrow samples

Bone marrow and peripheral blood samples have been collected before vaccination and during progression in heparinized tubes and mononuclear cells (MNCs) were isolated by Ficoll Isopaque density gradient centrifugation (Pharmacia, Germany).

### mRNA extraction and reverse transcription

Samples were resuspended in guanidium thiocyanate (GTC) buffer and stored at -80°C. Further processing of samples was performed as previously described [22]. In brief, total RNA was isolated by RNeasy Mini Kit including RNase-Free DNase Set (Qiagen, Germany) according to the manufactures recommendations. For reverse transcription, Omniscript Reverese Transcriptase kit (Qiagen, Germany) was used.

### Quantification of WT1 expression levels

Quantitative Real Time RT-PCR assays were performed using a LightCycler (Roche Diagnostics) with specific primers for WT1 and the housekeeping gene porphobilinogen deaminase (PBGD) as described elsewhere [22]. For quantification, PCR products generated from WT1 cDNAs and from porphobilinogen deaminase (PBGD) cDNAs were cloned into the vector pCR2.1-TOPO (Invitrogen, The Netherlands). A standard curve with 3 dilutions of the appropriate plasmid in duplicates was included in each PCR run. Analysis of RT-PCR expression data was performed with the LightCycler software (version 3). Crossing points were assessed by the second derivate maximum algorithm and plotted against the concentrations of the standards. Sample concentrations were calculated using the plasmid standard curve resulting in marker concentrations. All samples were analysed in duplicate. The average value of both duplicates was used as a quantitative value. To correct for differences of cDNA amount on a per-sample basis, results were provided as ratio to PBGD expression.

### Mutation analysis

Base exchanges within the epitope sequence or epitope flanking sequences (10 amino acids N- and C-terminal of the epitope) were analyzed with melting point analysis after amplification with the specific primers WT1 Mut fw 5'-TGTCCACTTTTCCGGC-3' and WT1 Mut rev 5'-GTCCCGTCGAAGGTGA-3 on a LightCycler instrument. To cover the whole sequence 2 wild-type complementary detection probe pairs were used (P, dephosphorylated; X, Fluorescein; Y, LC Red 640): probe pair 1: 5'-Y GCGCGTTAGGAAACATCCTGG P, 5'-TGGCCGGATGACGCCTGG X, probe pair 2: 5'-Y CTGGGCAGGTAGGGC P, 5'-TTAGGAAACATCCTGGCCTGGCCG X. To confirm the results obtained by melting curve analysis sequencing was performed in 4 patients.

### Flow cytometry

For determination of HLA Class I expression and HLA-A2 expression, leukemic blasts were stained with FITC conjugated anti-HLA class I monoclonal antibody (mAb) B9.12.1 (Beckmann Coulter) and with Alexa 647 conjugated mAb anti-HLA-A2 BB7.2 (AbSerotec) respectively. For exclusion of monocytes and lymphocytes samples were additionally stained with PerCP conjugated anti CD3 mAb and anti CD14 mAb (both BD Bioscience) and for exclusion of dead cells the LIVE/DEAD Fixable Violet Dead Cell Stain Kit (Molecular Probes) was used. For detection of WT1 expression in leukemic blasts extracellular staining was done with PE-conjugated mAb against CD34 (Becton Dickinson) and intracellular staining with mAbs against WT1 (clone 6F-H2, Dako) as primary antibody and goat anti mouse (GAM)-FITC (JacksonImmunoResearch) as secondary antibody. T cell response assessment was carried out as described in detail in Keilholz et al 2009 [8]. A cytokine response was considered positive if the percentage of WT1-peptide-specific cytokine producing CD3+CD8+T cells was at least 2-fold the percentage of cytokine producing CD3+CD8+T cells in response to an HIV control peptide; a tetramer response was considered positive if the frequency of tetramer positive CD3+CD8+ T cells exceeded 0.3%, which was the mean + 2 standard deviations (0.16% + 0.14%) observed in 12 healthy control subjects.

Data acquisition was performed on a FACSCalibur (Becton Dickinson) and data were analyzed using CellQuest software.

## Results and Discussion

Ten HLA-A2 positive patients with AML were analysed for mRNA expression levels of WT1 and for mutations of the WT1 epitope or epitope flanking sequences. All patients had received sequential vaccinations with the HLA-A2-restricted WT1 126-134 peptide with adjuvants within a phase II vaccination trial and progressed after an initial interval of vaccine efficacy [8]. T cell response to vaccination was analyzed as previously published in Keilholz et al 2009 (patient no 1; no 4; no 5; no 8; no 9; no 11; no 12; no 13; no 15; one patient is not published yet) [8]: In 8 of these 10 patients WT1 126-134 tetramer + T cells during the course of vaccination were found in peripheral blood (mean percentage of WT1 126-134 tetramer + cells in the CD3+ CD8+ T cell population 0.76% [0.3%-1.09%]). Moreover, to analyze the functional activity of WT1 126-134 specific T cells raised by vaccination, the reactivity of CD3+CD8+ T cells against WT1 126-134 peptide loaded cells was measured by intracellular IFN-γ and/or TNF-α cytokine staining. In 8 of 10 patients the presence of TNF-α and/or IFN-γ producing WT1 126-134 specific CD3+CD8+ T cells could be induced by vaccination. 7 patients showed a TNF-α response with a mean percentage of TNF-α+ cells in the CD3+CD8+ T cell population of 0.26% (0.1% - 0.6%) and 7 patients showed a IFN-γ response with a mean percentage of IFN-γ+ cells in the CD3+CD8+ T cell population of 0.23% (0.09-0.6%). However, one patient showed no WT1 126-134 tetramer + T cells or cytokine response in peripheral blood.

**Figure 1:**
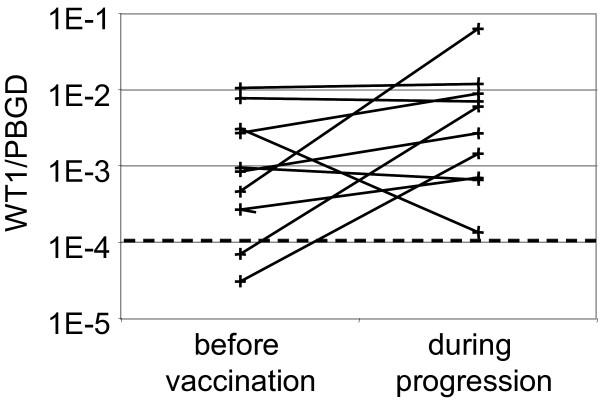
**WT1 expression levels before vaccination and during progression**. The relative amount was expressed as ratio WT1 [pg/μl]/PBGD [pg/μl]). Dotted line: normal bone marrow cut-off level.

As loss or downregulation of tumor antigens are potential immune escape mechanisms [12,13], first bone marrow samples obtained before vaccination and during progression were analyzed for expression levels of WT1 by real-time RT-PCR. In 9 out of 10 patients bone marrow WT1 levels were constant or increased at the time point of progression mirroring the kinetics of bone marrow blasts during treatment (figure 1). In one patient, however, down-regulation of WT1 by 1 log was observed although bone marrow blasts reached the same level at time of disease progression as before vaccination. In this latter patient WT1 protein was undetectable by intracellular flow cytometry at time of disease progression, consistent with downregulation of WT1 mRNA and protein as escape mechanism in this single patient.

WT1 mutations have been reported in about 10% of patients with AML [23]. They were frequently observed in DNA binding portions of the WT1 protein and may therefore contribute to leukemogenesis [24]. However, mutations have also been observed in exon 1, affecting the epitope flanking regions of the WT1 126-134 epitope [23]. Mutations of the epitope sequence or its flanking sequences could represent a possible immune escape mechanism as they may abolish proper HLA class I binding, T cell recognition or proteasomal processing. Therefore, mutation analysis was performed in both samples obtained before vaccination and during progression. However, no mutation leading to a base exchange within the epitope sequence or epitope flanking sequences could be detected in any patient. As expected, the known single nucleotide polymorphism C/T (NM_000378, mRNA position 790, coding position 3, protein residue Asn) at amino acid position 130 was observed in 6 patients.

To exclude failure of vaccine efficacy due to HLA class I downregulation [16-18,25], cell surface HLA class I expression on leukemic blasts was analyzed in 9 patients by flow cytometry during progression. The median percentage of HLA class I expressing blasts was 96% (84%-99%). Compared to HLA class I expression on blasts before therapy (5 patients analyzed) there was no significant downregulation. To exclude selective loss of the HLA-A2 allele, we analyzed HLA-A2 expression on leukemic blasts of 5 patients. In all 5 patients more than 90% of blasts stained positive for HLA-A2. In none of the patients a difference of HLA-A2 expression before therapy and at the time point of progression was observed.

## Conclusions

We have no evidence for an immune escape due to loss or mutation of WT1 or HLA class I downregulation as has been reported for immunotherapy targeting differentiation antigens in melanoma [12]. This finding supports the use of tumor target antigens like WT1 which are crucial for tumor cell proliferation. However, further studies, especially on mechanisms of immune evasion at the effector phase of the anti-tumor immune response, are indicated to determine potential inhibitory immune mechanisms during WT1 peptide vaccination.

## Competing interests

Supported by a grant from the from the José-Carreras Leukemia Foundation and from the "Stiftung zur Bekaempfung der Leukaemie"

## Authors' contributions

AB has made substantial contributions to conception and design, acquisition of data, analysis and interpretation of data and wrote the manuscript; AL, AN and OS have made substantial contributions to acquisition of data, analysis and interpretation of data. CS have been involved in conception and design, interpretation of data and revising the manuscript critically for important intellectual content, ET has made substantial contributions to conception and design and was involved in revising the manuscript critically for important intellectual content, UK: has made substantial contributions to conception and design, as well as analysis and interpretation of data and wrote the manuscript.

All authors have read and approved the final manuscript.
